# Stiffness and Aging in Cardiovascular Diseases: The Dangerous Relationship between Force and Senescence

**DOI:** 10.3390/ijms22073404

**Published:** 2021-03-26

**Authors:** Silvia Ferrari, Maurizio Pesce

**Affiliations:** 1 Unità di Ingegneria Tissutale Cardiovascolare, Centro cardiologico Monzino, Istituto di Ricovero e Cura a Carattere Scientifico(IRCCS), 20138 Milan, Italy; maurizio.pesce@ccfm.it; 2 PhD Program in Translational Medicine, Department of Molecular Medicine, Università degli studi di Pavia, 27100 Pavia, Italy

**Keywords:** senescence, aging, mechano-transduction, epigenetics

## Abstract

Biological aging is a process associated with a gradual decline in tissues’ homeostasis based on the progressive inability of the cells to self-renew. Cellular senescence is one of the hallmarks of the aging process, characterized by an irreversible cell cycle arrest due to reactive oxygen species (ROS) production, telomeres shortening, chronic inflammatory activation, and chromatin modifications. In this review, we will describe the effects of senescence on tissue structure, extracellular matrix (ECM) organization, and nucleus architecture, and see how these changes affect (are affected by) mechano-transduction. In our view, this is essential for a deeper understanding of the progressive pathological evolution of the cardiovascular system and its relationship with the detrimental effects of risk factors, known to act at an epigenetic level.

## 1. Chronological Aging vs. Biological Aging

Biological aging, also called functional or physiological aging, is associated with a gradual decline in several physiological functions. Typically, this phenomenon heterogeneously affects multiple-organ systems, leading to progressive tissue damage and dysfunction. Aging is an unmodifiable risk factor for the onset of different pathologies, such as neurodegenerative disorders, cardiovascular diseases, cancer, and diabetes [1]. Cellular senescence is one of the hallmarks of biological aging and is characterized by a permanent cell cycle arrest in which cells become resistant to growth-promoting stimuli and undergo metabolic, morphological, and chromatin changes with modification in gene expression [2].

To date, there are various molecular mechanisms accounting for the onset of cellular senescence. These include exposure to reactive oxygen species (ROS), telomere shortening, and chronic inflammation. ROS, mainly produced in the mitochondria, are chemically unstable molecules whose levels increase with age, thereby damaging DNA, proteins, and cells membranes [3]. It has been reported that mitochondria in senescent cells showed increased concentrations of ROS, and thus indicated mitochondrial dysfunction [4]. Moreover, studies in *Drosophila melanogaster* and *Caenorhabditis elegans* demonstrated that the overexpression of enzymes with ROS-scavenging activity resulted in a significant extension of lifespan [5]. Telomeres are repetitive DNA sequences protecting the chromosome ends from end-to-end fusions and degradation [6]. Telomeres shorten at each cell division and, below a critical length, cause cellular senescence. The existence of a critically minimal telomere length was first reported in the early 1960s by Leonard Hayflick. By studying human fibroblasts isolated from embryonic lung tissues, he showed that cells stopped proliferating after a limited number of population doublings, the so-called Hayflick limit. The length of the telomere at birth is about 11 to 15 kb, while in the elderly it is significantly shorter (≃4 kb) [7].

Besides the concept of functional aging, it is important to define chronological aging, which simply indicates the time gone by since birth, and is not affected by individual lifestyle or environment. Commonly, biological age is thought to equate to chronological age. On the other hand, it has been demonstrated that the genome may exhibit signs of accelerated (biological) aging depending on the occurrence of methylation at specific CpG sites (regions with a large number of Cytosine-Guanine dinucleotide repeats) [8]. According to this, the rate of biological aging may not be the same in all individuals with the same chronological age, and the two types of aging may not be necessarily in accordance [9]. An example of how chronological and biological aging do not always coincide is the Hutchinson–Gilford progeria syndrome. This rare pathology is characterized by premature aging in children from early infancy, and is associated with dramatically accelerated cardiovascular disease leading to death from myocardial infarction or stroke between the ages of 7 and 20 years. Progeria syndrome is caused by a mutation in lamin A, a central component of the inner nuclear lamina involved in chromatin organization and gene transcription [10]. In this review we will discuss the effects of biological aging on tissue structure, subcellular organelles’ reorganization, nucleus architecture, and modifications of the surrounding extracellular matrix (ECM), in the emerging view that the senescence of cells inside aging tissues may have connections with altered mechanical sensing and vice versa.

## 2. ECM Main Components Change with Aging and Affect Mechano-transduction

The ECM consists of two main classes of macromolecules: proteoglycans and fibrous proteins. Proteoglycans are hydrophilic molecules essential for hydrogels’ formation, which enable matrices to withstand high compressive forces. Fibrous proteins, such as collagen, elastin, fibronectins, laminin, and the enzymes deputed to their synthesis and degradation, represent crucial players in ECM remodeling [11]. Collagen, the primary structural element and the most abundant protein in the ECM, provides tensile strength to tissues [12]. Either fibroblasts in the stromal matrix or those recruited from the neighboring tissues synthetize and organize collagen fibers. These cells exert tensioning forces on the matrix and organize collagen fibrils, determining the alignment of the fibers [13]. Modifications in collagen synthesis, and thus altered ECM stiffness, represent some of the main features of aged tissues, in which cells show abnormal orientation, migration, and alignment. The hypothesis that the synthesis of collagen is responsible for the stiffening of the ECM and that this increases with aging was first proposed by Alnaqeeb and colleagues [14]. Along the same line, Debessa and colleagues, analyzing human autopsies, demonstrated that the amount of collagen increased from 3.9 ± 0.8% in 20–25-year-old individuals to 5.9 ± 0.8% in 67–87-year-old individuals [15]. Moreover, it has been reported that collagen I levels were higher in the hearts from autopsies of 80-year-old patients compared to younger ones. Since collagen I has a high tensile strength, its age-related increase affects cardiac biomechanics. Collagen structure in the ECM is mainly stabilized by cross-link formation by lysyl oxidase (LOX)-mediated aldehyde between the lysine or hydroxylysine residues. There is evidence that cross-linking, measured by hydroxylysyl pyridinoline concentration, significantly increases with age and alters myocardial stiffness [16]. As the amount of collagen in the ECM increases, LOX enhances its cross-linking activity, resulting in the formation of mature collagen fibers resistant to metallo proteinases (MMPs), and thus a higher local elastic modulus [17]. The stiffening of the ECM might compromise cardiomyocytes’ mechano-sensing properties, likely leading to cardiac dysfunctions [18]. In addition, the matricellular protein secreted protein acidic and rich in cysteine (SPARC), expressed in cardiac fibroblasts, is involved in collagen cross-linking and promotes the deposition of collagen in the myocardium [19]. Studies have demonstrated that the depletion of SPARC prevented age-related collagen increase in mice [20]. Therefore, LOX and SPARC play a crucial role in myocardial stiffening with aging.

Another main component of the ECM in the cardiovascular system is fibronectin (FN), a glycoprotein involved in matrix organization with a crucial role in cell adhesion, migration, and spreading [21]. Forces exerted by resident fibroblasts induce the unfolding of FNs that then expose binding sites, hidden in the relaxed status, resulting in altered cellular behavior and mechano-sensing properties [22]. Moreover, it has been demonstrated that FNs’ mRNA levels, as well as proteins, increase significantly during the process of cellular aging in human fibroblasts with consequences on cell spreading [23]. Similar to data previously reported for collagen, an increase in FN synthesis with age might be involved in the stiffening and thickening of vasculature, leading to cardiovascular diseases (CVDs). The downstream machinery of FN signaling through integrins is also affected by aging. For example, the overexpression of integrin-linked kinase (ILK), a cytoplasmic protein regulating FN–matrix assembly, has been associated with aging and the accumulation of FN in tubular epithelial cells from rats [24]. Chen and colleagues demonstrated that young cardiac fibroblasts overexpressing ILK showed senescent phenotypes (i.e., flattened cell shapes, higher levels of SA-β-Gal-positive cells) and modifications in FN organization. On the other hand, the depletion of ILK in old cells attenuated senescent cell-like modifications mentioned before. Taken together, these data suggest that changes in ILK levels could significantly affect not only cytoskeleton organization, but also the level of cellular senescence involving a possible crucial role of FN signaling [25].

## 3. Mechanical Changes in Aging Cytoskeleton

One of the hallmarks of the aging process is the increase in cell stiffness and the cells’ loss of ability to promptly rearrange their cytoskeleton in response to stimuli. The mechano-transduction pathway in senescent cells is, therefore, less effective [26]. Furthermore, mechanical properties of the lipid bilayer undergo oxidative modification, which is one of the main features of aging [27]. These modifications include polysaturated fatty acids and cholesterol–lipid layers, which are stiffer and thicker compared to those with polyunsaturated fatty acids. Products of lipid oxidation and an increase in cholesterol levels, indeed, are commonly considered as markers of cells’ senescence [28]. Lieber and colleagues performed atomic force microscopy (AFM) on human cardiac myocytes and demonstrated a significant increase in the apparent elastic modulus of single, aged cardiac myocytes [29]. The same correlation between stiffness and aging was found in human fibroblasts and epithelial cells [30]. The relationship between the change in mechanical properties of cells, aging, and modification in the composition of the plasma membrane has been shown in red blood cells. Indeed, it was demonstrated that cell rigidity increases with age, with a corresponding decrease in cell deformability, as a result of lipid peroxidation and increased levels of cholesterol [28]. High levels of cholesterol may be, in turn, associated with an increase in plasma cholesterol [31] or with age-dependent oxidative stress [32].

Despite the evidence regarding the stiffening of the aged matrix or cellular membrane in several cell types, there are also clues showing a softening of the cytoplasm with age. For example, Zahn and colleagues measured the average Young’s moduli of old and young human skin fibroblasts, demonstrating that the former were softer and had lower amounts of actin. Moreover, older cells stimulated with uniaxial tensile strain showed a significantly faster reorganization [33]. These contrasting data may be due to the effect of cell heterogeneity influenced by several factors including genetic predisposition, individual lifestyle (smoking, diet, exercise), or environmental impact [34]. Since cell-based mechano-transduction is essential for physiological cellular functions, the stiffening of senescent cells is often associated with the onset of pathologies such as cancer, vascular degeneration, and cardiac dysfunction [35,36]. With this in mind, it is important to unravel the molecular pathways leading to the loss of cells’ functionality. Given the crucial role of the cytoskeleton in mechano-transduction, we will discuss the age-related changes of its main components (i.e., actin, microtubule, intermediate filaments).

Filamentous actin (F-actin) is critical for stress responses and also mediates ECM nuclear–mechanical coupling [37]. Cheung et al. investigated whether the senescence of human endothelial cells (ECs) is associated with an increase in traction forces through modification in the antioxidant regulator deacetylase Sirtuin1 (SIRT-1), commonly downregulated during aging [38]. The authors found that traction forces were higher in aged ECs, and this increase correlated with abnormal actin localization. Moreover, these cells showed stiffer actin filaments. The depletion of SIRT-1 had no effect on the actin structure of younger cells, while its activation reduced traction forces and increased the peripheral actin in old cells. These results clearly demonstrate that ECs’ senescence increases traction forces and alters actin localization through levels of SIRT-1. A similar increase in actin stiffness was reported in fibroblasts from aged compared to young donors [39] and in old, murine T cells [40]. In particular, it has been recently demonstrated that CD4 T cells from old mice show architectural cytoskeletal modifications that are not dependent on alterations in the T-cell receptor (TCR) signal transduction. These changes included, besides the previously mentioned higher level of F-actin polymerization, a lack of lamellipodia and an increase in CD4 T cell membrane association to the cytoskeleton. These data led the authors to hypothesize an interesting “immuno-senescence” model in which aging leads to an increase in the activity of Vav, a GTPase crucial for F-actin formation and cytoskeletal remodeling during TCR signaling, resulting in an increased F-actin level in CD4 T cells from old donors. These higher levels of F-actin might be responsible for a decrease in membrane fluidity with a drastic impact on TCR signaling. This evidence confirms the hypothesis that the stiffer phenotypes observed in older individuals may be caused by abnormal actin polymerization.

Vinculin, a component of the intracellular focal adhesion complex, is a force-sensitive cell shape regulator and an intracellular signaling transducer molecule. This cytoskeletal protein is involved in the anchoring of integrins to the actin cytoskeleton [41]. Using a *D. melanogaster* aging model system, Kaushik and colleagues found a correlation between the cardiac vinculin overexpression and an extensive cytoskeletal reorganization, associated with enhanced cardiac contractility and life span extension [42]. In this study, in contrast to the idea that remodeling during aging is mainly maladaptive, the increasing levels of vinculin have a protective effect in time, providing resistance to mechanical stress and improving myofilament organization. The beneficial role of this mechano-sensing protein has been confirmed in mouse and rat models: cardiac-specific depletion of vinculin led to sudden death from ventricular tachycardia, while survivor mice died from cardiomyopathy within 6 months [43]. Although of particular interest for novel therapeutic approaches, the molecular mechanisms responsible for senescence-associated vinculin overexpression are still unclear.

Microtubules (MTs) are ubiquitous intracellular structures involved in the control of several functions, including cell division, regulation of shape and polarity, and intracellular transport [44]. Moujaber et al. demonstrated that in renal proximal tubule cells, microtubule stability increased during senescence, providing a higher resistance of the filaments against disassembling forces [45]. This may be in part due to the loss of Rock1 kinase, a regulator of proper MT organization. The authors hypothesized that alterations of MTs’ dynamics might have a drastic impact on senescent cells, resulting in their failure to form centrosomes, to migrate, and to proliferate. This mechanism could be involved in the onset of several diseases, since the inability of cells to migrate properly has a severe effect on the replacement of injured cells in organs. Modifications in MTs’ network stability and density are indeed one of the major markers of pathological cardiac remodeling, since they are able to block cardiomyocytes’ contractile function leading to several CVDs, including hypertrophy and heart failure. Moreover, the role of MTs in mechano-transduction becomes essential for the acute and chronic adaptation of the cardiovascular system to changing loads [46]. Despite the importance of MTs, it is necessary to investigate further how they are able to maintain and shape the membrane of cardiomyocytes.

## 4. The Impact of Senescence-Related Nuclear Modifications on Cell Mechanical Response and Transcription Activity

Although conventionally believed to be a rather static structure, the cell nucleus is a specialized and dynamic organelle continuously exposed to mechanical stimuli. Aging-associated nuclear alterations have drastic impacts on nuclear architecture and on the chromatin structure, resulting in gene expression level changes that compromise tissue function [47]. The nuclear lamina (NL) is composed of nuclear membrane-associated and fibrous proteins that form a scaffold at the nucleus periphery. This structure not only gives mechanical support to cells, providing an integrated connection between the cytoskeleton and the nucleus, but also is involved in the regulation of gene expression levels [48]. The most abundant proteins of the NL are lamin A, lamin B1, lamin B2, and lamin C, contributing to the nucleus’ structural support and stiffening. These molecules are essential for the transduction of mechanical cues from the extracellular environment to the nucleus and may be altered in the senescence process [49]. Recent studies have demonstrated that nuclear shape and ECM stiffness are able to regulate gene expression by exposing chromatin to mechanical stress, likely through a direct/indirect reshuffling of transcription factors’ accessibility [50]. In the presence of mechanical cues, indeed, forces are transduced to chromatin by the actomyosin stress fibers of the cytoskeleton, anchored to the linker of nucleoskeleton and cytoskeleton (LINC) complex and nuclear lamins. As a consequence, chromatin stretches, altering its accessibility to RNA polymerase II and thus impacting cell transcriptional activity. This event might be involved in the onset of several CVDs; for example, myofibroblast pathological activation could be the result of the mechanical stimuli transduced in the nucleus by the cytoskeleton, thereby activating pro-fibrotic signaling [51].

Evidence shows that lamin B1 (LB1) expression is tightly associated with the development of aging disorders, since its reduction has been reported in age-related phenotypes, e.g., progeria syndrome [52]. Shimi and colleagues demonstrated that in human lung embryonic fibroblasts, LB1 has a key role in cell senescence. Indeed, this protein decreases dramatically as cells become senescent, and its depletion resulted in premature senescence [53]. In primary human and murine cell strains, the induction of senescence by DNA damage, replicative exhaustion, or oncogene expression resulted in lamin B1 loss. However, another study demonstrated that the depletion of lamin B1 in human fibroblasts resulted in an increase in senescent cells only in the presence of additional stress (low-density vs. subconfluence seeding conditions). Data showed that only cells seeded at a low density presented a higher level of senescence, suggesting that ablation of lamin B1 is not itself enough to trigger senescence [54]. In the same study, the authors attempted to investigate the cause of the impairment in the proliferation of lamin B1-depleted cells and, by analyzing cell cycle profiles, they found an increase in the percentage of cells in Growth phase-2 (G2)/Mitosis phase (M). This, together with the chromosomal instability reported in other models, finally leads to impaired chromosome segregation and karyotype abnormalities [55] with a consequent mitotic block. Finally, it has been reported that lamin gene defects occur in 33% of the cases of dilated cardiac myopathy with atrioventricular block. In these patients, myocytes’ nuclei were severely damaged, probably leading to cell death and gene expression alterations [56]. Abnormalities in lamin-A/C assembly and its interaction with another nuclear protein, Emerin, might finally cause the altered organization of nuclear actin and the nucleocytoplasmic shuttling of MKL1, a mechano-sensitive transcriptional factor crucial in cardiac development and function. 

## 5. Epigenetic Modifications in Chromatin Architecture and Aging

**Figure 1:**
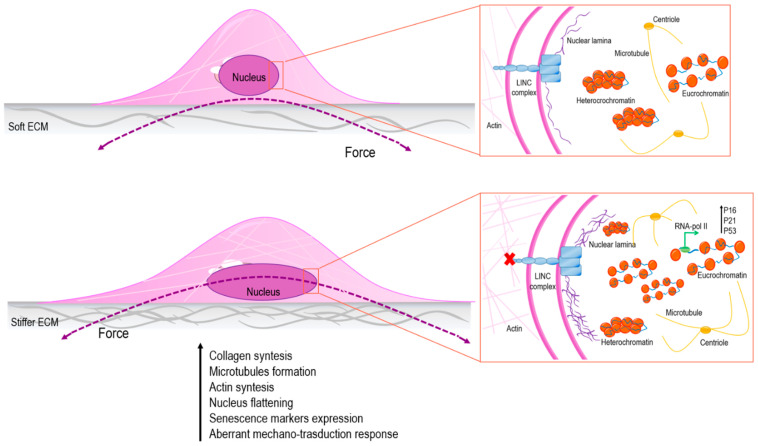
Stiffness of the nucleus affects structure and organization of chromatin with consequences on gene transcription. In particular, in the presence of soft extracellular matrix (ECM), the cell nucleus is subject to low straining forces and the amount of heterochromatin is higher than euchromatin; on the other hand, in presence of stiffer ECM, the cell nucleus is stretched, and, as a result, chromatin changes its status into a more relaxed conformation, promoting senescence markers’ expression [59,60,61].

Epigenetics refers to a series of DNA/chromatin modifications that do not affect the primary sequence of DNA. Epigenetics plays a central role in the control of genome functions through different covalent chromatin modifications (i.e., DNA methylation, histone modifications, interaction with noncoding RNA) that alter the architecture of chromatin and, as a result, DNA’s accessibility to transcriptional factors [57]. Growing evidence demonstrates that epigenetic modifications are involved in cell senescence, and, since the structure and organization of chromatin influences the mechanics of the nucleus, this might have important readouts in a mechano-transduction perspective [58]. As mentioned previously, forces generated by cytoskeleton tensioning are transferred from focal adhesions to the nucleus through the LINC complex and the nuclear lamins. As a result, the chromatin stretches and its accessibility to RNA polymerase II changes. It has been demonstrated that the LINC complex, due to the loss of its components, is altered during senescence, and this could affect how force is transmitted to the nucleus, resulting in an aberrant cellular mechano-response [59]. Emerging data show that ECM stiffness may also affect nuclear architecture and, consequently, modify chromatin organization through a direct connection of the nuclear lamina with the contractile cytoskeleton network [60]. For instance, Discroll and colleagues demonstrated that the inhibition of stress-fiber contractility by pharmacological actin depolymerization significantly decreases strain transfer to the nucleus. This, in turn, was reflected in nucleus deformation and, thus, on transcription factors’ translocation through the nuclear pores [60,61]. Moreover, in the presence of stiffer ECM, chromatin was dislodged from the nuclear periphery and changed its conformation to a relaxed status (euchromatin), which facilitated gene transcription [62]. In particular, in the context of senescence, the higher amount of euchromatin may be responsible for the transcription of senescence-related genes (Figure 1). In confirmation of these results, unpublished data from our group reveal that pharmacological interference with F-actin polymerization affected not only the dynamic deformation of the nucleus, but also the shuffling of the transcriptional factor Yes-associated protein (YAP) in cardiac stromal cells. Swift and colleagues demonstrated that matrix stiffness directly increased lamin A protein levels, which stabilized the nucleus, and this was associated with higher histone H3-Lysine9-trimetilation (H3K9m3) levels on chromosomes [63]. These data are of particular interest since abnormalities in lamin A assembly occur in age-related pathologies like progeria syndrome. In keeping with this evidence, Jain and colleagues used a mouse fibroblast model to evaluate the actomyosin-dependent nucleocytoplasmic shuttling of transcription and epigenetic factors histone deacetylase-3 (HDAC3), and MKL [61]. Cells seeded on small adhesive areas showed a decrease in nuclear volume and higher histone acetylation levels. Moreover, the lower actomyosin contractility caused HDAC3 nuclear shuttling, leading to a reduction in histone acetylation levels and the consequent chromatin compaction with reduction in nuclear volume. In another study on mice fibroblasts, authors demonstrated that treatment with inhibitors of the actomyosin fibers’ contractility, Blebblistatin and Y27632, resulted in the degradation of IκB-α (a nuclear factor that normally interferes with HDAC3 nuclear translocation). IκB-α degradation released the acetylating enzyme that shuttled into the nucleus. Taken together, these data show an interesting correlation between the actomyosin contractility and the nuclear translocation of the epigenetic factors, with important consequences on the cellular transcriptional activity. As described previously, nuclear structure alterations are recurrent in aging, so this could also have consequences on epigenetic mechanisms.

A final example of the translation of cell mechanics into epigenetic phenomena is the shear stress (SS), defined as the frictional force generated by blood flow on the endothelial surface. SS is responsible for the activation of endothelial cells (ECs), leading to the transcription of specific genes. These genes include mechano-sensory complexes including the platelet–endothelial cell adhesion molecule 1 (PECAM-1) and vascular endothelial cadherin (VE-cadherin) [64]. In turn, the expression of these receptors is regulated by changes in chromatin organization depending on the activity of histone acetyltransferases (HATs) and histone deacetylases (HDACs) [65]. Interestingly, SS induces the expression of the gene encoding for endothelial nitric oxide synthase (eNOS) gene expression through the activity of HDACs, and eNOS activates telomerase, thus retarding endothelial cell senescence [66].

Generally, there are several age-related recurring histone modifications, such as an increase in the trimethylation of H4K20 and H3K4 and a decrease in the methylation of H3K9 and H3K27 [67] (Table 1). On the other hand, other epigenetic modifications have been associated with an increase in life span, such as a lower level of global DNA methylation [68]. Since DNA methylation is responsible for the formation of constitutive heterochromatin, its reduction leads to a more relaxed chromatin organization. This implies a lower nuclear compliance and a higher cell transcriptional activity. Furthermore, growing evidence shows that there is a correlation between heterochromatic structure that accumulates in senescent human fibroblasts and senescence [69]. These chromatin accumulations, known as senescence-associated heterochromatic foci (SAHF), promote cell cycle arrest and gene expression repression.

Of particular interest in the context of CVDs are, finally, epigenetic modifications occurring in cardiomyocytes with aging. Using novel multi-omics technologies, investigators have analyzed cardiomyocyte-specific global histone modification profiling in failing human hearts. The authors found that pathological gene expression was associated with histone-activating marks (H3K9ac, H3K27ac, H3K4me3) and repressive marks (H3K27me3) [70].

In conclusion, the possible interaction between mechano-transduction pathways and epigenetics may play a central role in the regulation of gene expression levels involved in the loss of cell function, which is typical of the senescence status.

## 6. Future Perspectives: Phenotypical Reversion of Senescent Cells 

Given the impact of age-dependent cellular and tissue mechanical dysfunctions on health, a growing question is whether it is possible to revert the phenotype of senescent cells. Since one of the main modifications occurring during aging is the increase in F-actin levels from different cellular types, the administration of drugs interfering with F-actin polymerization may represent an effective strategy. In this regard, it was demonstrated that the treatment of senescent human fibroblasts with cytochalasin B [76], an F-actin-depolymerizing drug, resulted in a decrease in cytoplasm stiffness comparable to younger cells’ values. Senolytic drugs are also emerging as a promising therapy; this class of drugs selectively targets and clears senescent cells accumulating due to mechanical stress, DNA damages, and reactive metabolites [77]. In a model of aged mice, the intermittent administration of Dasatinib and Quercetin enhanced cardiac and vascular functions with a reduction in vessels’ calcification, a decrease in senescence markers, and a partial reversion of cardiomyocyte hypertrophy or left ventricular fibrosis [78,79].

Since epigenetics have a crucial impact on senescence and several epigenetic modifications are reversible, another novel therapeutic approach might be the employment of epigenetic drugs. For example, the inhibition of both DNA methylation and histone deacetylation by combined treatment of 5-azacytidine and valproic acid resulted in an increased plasticity and function of human endothelial progenitor cells, with beneficial effects on myocardial repair [80]. Moreover, the use of the histone acetylase (HAT) activator pentadecylidenemalonate 1b (SPV106) in cardiac mesenchymal cells (CMSC) obtained from type 2 diabetic patients (D-CMSC) was able to restore proliferation and differentiation to levels observed in cells derived from normoglycemic subjects. In addition, histones and DNA methylation levels were reduced, and senescence was also significantly inhibited [81]. Another promising therapy with epigenetic-active drugs is the use of HDAC inhibitors (HDACi). For example, the pan-HDACi suberoylanilide hydroxamic acid (SAHA) reduced infarct damage and attenuated pathological cardiac remodeling in rodent and rabbit models of myocardial infarction (MI). Valproic acid (VPA), a branched short-chain fatty acid that reduces class I and class IIa HDACs’ activity [82], was found to protect the heart through the Foxm1 (a transcriptional activator involved in cell proliferation) pathway after acute MI [82]. Moreover, the administration of Trichostatin A, a broad-spectrum HDACi, in cultured embryonic stem cells promoted myogenesis and angiogenesis and minimized the loss in myocardial functionality after MI [83]. Finally, the inhibition of CK2α1, an enzyme that activates HDAC-2, showed similar beneficial effects to HDAC inhibitors in the reduction of cardiac hypertrophy [84]. Thus, besides the use of HDAC inhibitors, which are generally regulated by post-translational modifications, the use of drugs targeting HDAC-regulating enzymes may be effective.

## 7. Conclusions 

This review highlights the dangerous interplay of cellular mechano-transduction and aging as a possible determining factor of the early programming and progression of cardiovascular diseases. Although senolityc, epigenetic, and cytoskeleton-interfering drugs represent a promising and novel therapeutic approach, it is necessary to proceed with a deeper understanding of the molecular pathways, potentially establishing the tight association between cell-based mechano-sensing and cellular senescence. Novel interdisciplinary approaches integrating measurements of the forces actually developed by the cells [85] along with determination of the nuclear topology [86] and chromatin rheological features [87] will open novel avenues to resolve the problem of cellular senescence and retard the onset of aging-related diseases.

## Figures and Tables

**Table 1:** Histone modification patterns in association with aging.

Modification	Change	Role in Transcription	References
H3K4me2	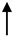	Repression	[71]
H3K4me3	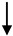	Activation	[70]
H3K9me	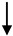	Activation	[67]
H3K9me2	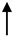	Repression	[72]
H3K9me3	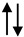	Activation/Repression	[72]
H3K9Ac	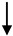	Activation	[70]
H3K27Ac	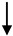	Activation	[70]
H3K27me3	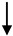	Repression	[73]
H3K56Ac	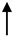	Activation	[72]
H420me	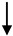	Repression	[72]
H420me2	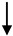	Repression	[74]
H4K20me3	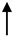	Repression	[64]
H416ac	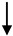	Repression	[75]

↑Increase; ↓reduction.

## Data Availability

Not applicable.
